# Estimating fish abundance at spawning aggregations from courtship sound levels

**DOI:** 10.1038/s41598-017-03383-8

**Published:** 2017-06-13

**Authors:** Timothy J. Rowell, David A. Demer, Octavio Aburto-Oropeza, Juan José Cota-Nieto, John R. Hyde, Brad E. Erisman

**Affiliations:** 1 Marine Biology Research Division, Scripps Institution of Oceanography, University of California San Diego, 9500 Gilman Drive, La Jolla, California 92093 USA; 2 NOAA Fisheries, Southwest Fisheries Science Center, 8901 La Jolla Shores Drive, La Jolla, California 92037-1508 USA; 3 Centro Para la Biodiversidad Marina y Conservación A.C., Calle del Pirata, # 420, Fracción Benito Juárez, La Paz, Baja California Sur 23090 Mexico; 4 Marine Science Institute, The University of Texas at Austin, 750 Channel View Drive, Port Aransas, Texas 78373 USA

## Abstract

Sound produced by fish spawning aggregations (FSAs) permits the use of passive acoustic methods to identify the timing and location of spawning. However, difficulties in relating sound levels to abundance have impeded the use of passive acoustics to conduct quantitative assessments of biomass. Here we show that models of measured fish sound production versus independently measured fish density can be generated to estimate abundance and biomass from sound levels at FSAs. We compared sound levels produced by spawning Gulf Corvina (*Cynoscion othonopterus*) with simultaneous measurements of density from active acoustic surveys in the Colorado River Delta, Mexico. During the formation of FSAs, we estimated peak abundance at 1.53 to 1.55 million fish, which equated to a biomass of 2,133 to 2,145 metric tons. Sound levels ranged from 0.02 to 12,738 Pa^2^, with larger measurements observed on outgoing tides. The relationship between sound levels and densities was variable across the duration of surveys but stabilized during the peak spawning period after high tide to produce a linear relationship. Our results support the use of active acoustic methods to estimate density, abundance, and biomass of fish at FSAs; using appropriately scaled empirical relationships, sound levels can be used to infer these estimates.

## Introduction

Quantitative assessments of fish abundance form the basis of fisheries management and conservation strategies^1, 2^, but the complex migratory patterns, broad spatial distributions, life histories, and population dynamics of many fish species make it difficult to survey entire populations^3^. Some of these challenges are minimized if surveys are conducted where and when fish form large conspecific spawning aggregations^4^. Fish spawning aggregations (FSAs) occur at predictable times and locations, permitting surveys to estimate spawning stock abundance, biomass, and length and age distributions of regional populations^5, 6^.

Efforts to monitor FSAs and manage fishing intensity often rely on fisheries-dependent data (e.g., catch per unit effort) to estimate stock abundance^7^. However, these data can be insensitive to population declines since fish densities at FSAs may not change proportionally with abundance^8, 9^, making these methods inefficient for monitoring, assessments, and establishing quotas of total allowable catch. Fisheries-independent data often yield more accurate assessments of stocks^10^, but methods such as mark-recapture, visual census, and trawls can be invasive, laborious, expensive, and inefficient or ineffective across the diverse environments where FSAs occur^11, 12^. Therefore, the continued development and expansion of fisheries-independent methods to survey FSAs in challenging habitats are greatly needed to improve the quality of population assessments and manage vulnerable spawning stocks in a sustainable manner^6^.

The adaptability of active acoustic methods to survey fishes in both deep^13^ and shallow water habitats^14, 15^ enables assessments of populations across a spectrum of environments where constraints of depth and low visibility prevent the use of other methods. As a trusted source of fisheries-independent data for resource managers and policy makers^16, 17^, active acoustic surveys have been used to estimate abundance, biomass, and length and spatial distributions of fish stocks at FSAs^13, 18, 19^. However, the cost and complexity of collecting and processing data have impeded the widespread application and long-term use of active acoustics for assessments of most aggregate spawning fishes, which are often associated with small-scale fisheries in developing countries^6^.

Increased recognition of sound production in over 100 families of marine fishes^20, 21^ has prompted an insurgence of passive acoustic methods to non-invasively and efficiently study and monitor populations^22–24^. As many fishes produce species-specific calls associated with courtship and reproduction, recordings of sound production have been used to identify the timing and location of spawning in a number of exploited species, such as Atlantic Cod^18^ (*Gadus morhua*), Pollock^25^ (*Pollachius pollachius*), Haddock^26^ (*Melanogrammus aeglefinus*), Nassau Grouper^27^ (*Epinephelus striatus*), and numerous species of croakers^28, 29^ (family Sciaenidae). While some progress has been made to use received sound levels as indices of fish presence, behaviors, and relative abundance at FSAs^30–34^, difficulties in relating sound production metrics to fish densities have precluded passive acoustic estimations of abundances for use in quantitative population assessments^22, 23, 35^.

With an understanding of fish calling rates and sound propagation, it is possible to estimate abundance from call occurrences^22, 35, 36^; however, call rates at FSAs often vary considerably^29, 37, 38^ and are difficult to characterize in-situ for fishes. Additionally, fish choruses at some FSAs prevent the isolation of individual calls^29^. A more tractable approach may be to utilize relationships between sound production levels, measured using passive acoustics, with independent measures of fish density estimated with active acoustics^22, 35^. Ultimately, species-specific models could be used to assess and monitor spawning stock densities, abundances, and biomasses using passive acoustic methods during periods with stable rates of sound production^33, 39, 40^.

Croakers are an ideal assemblage of fish species to develop and test relationships between sound production and independent measurements of fish density at FSAs, because they produce sounds during spawning activities^41, 42^ within FSAs that form in estuaries and coastal habitats^43, 44^ (Table 1). Also, croaker fisheries generate tens of billions of US dollars globally each year^45, 46^, and among the 166 species of croakers for which information is available on the IUCN Red List, those that are threatened or endangered are aggregate spawners that are also overfished^47, 48^. Therefore, new methods that demonstrate the use of passive acoustic technologies to estimate density and assess spawning populations of croakers will support the development of monitoring programs that aid adaptive management strategies to prevent stock declines, hasten recovery, and support sustainable harvest of other vulnerable, aggregating species that produce sound during spawning (Table 1).

**Table 1:** Families of commercially important fishes that have been documented to produce sounds and form spawning aggregations.

Family	Common Names	No. Species	Total Landings (tons)
Clupeidae	Herrings, Shads, Sardines, Menhadens	198	9,087,812
Gadidae	Cods and Haddocks	24	6,637,665
Carangidae	Jacks and Pompanos	146	4,315,926
Sciaenidae	Croakers, Drums, and Weakfishes	283	1,908,360
Sparidae	Porgies	150	439,887
Epinephelidae, Serranidae	Groupers and Sea Basses	537	343,236
Lutjanidae	Snappers	110	268,976
Sebastidae, Scorpaenidae	Rockfishes, Rockcods, and Thornyheads	132, 216	265,410
Mullidae	Goatfishes	87	220,604
Haemulidae	Grunts	133	121,682
Ophidiidae	Cusk-eels	258	41,353
Labridae	Wrasses	520	23,464
Ictaluridae	Catfishes	51	12,733
Acanthuridae	Surgeonfishes, tangs, unicornfishes	82	9,785
Scaridae	Parrotfishes	100	5,266
Acipenseridae	Sturgeons	25	422
Batrachoididae	Toadfishes	83	339
**Total**		**3,135**	**23,702,920**

The table presents total global landings (tons) reported in 2013 (FAO) for aggregating, sound producing families of fishes. Families are organized by total landings. Sciaenidae, ranking fourth in contributions to landings, supports global economies and is need of new assessment methods to ensure sustained productivity.

**Figure 1:**
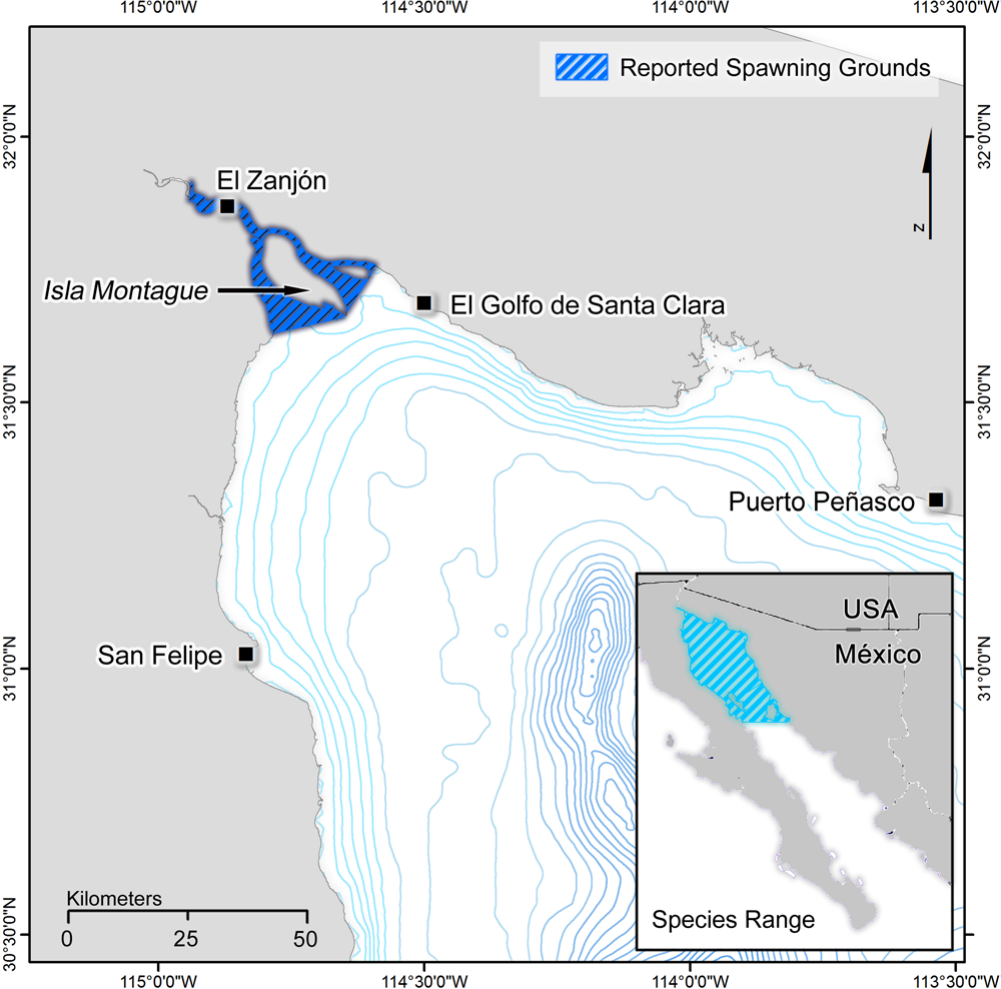
Species range and spawning grounds of Gulf Corvina (*Cynoscion othonopterus*). The species range extends from the Midriff Islands to the northern limit of the Gulf of California (inset map). During the months of February to early June, the reproductive population migrates northward to the only reported spawning grounds in the Colorado River Delta. The map is adapted by permission from Macmillan Publishers Ltd: [SCIENTIFIC REPORTS] (Erisman *et al*.^43^), copyright 2012.

In this paper, we developed active and passive acoustic methods to assess the abundance of Gulf Corvina (*Cynoscion othonopterus*; family Sciaenidae), hereafter Corvina, at its FSA and constructed a model to quantify relationships between sound levels and fish density during spawning. From late February to early June, during the outgoing tide over 2–3 day periods prior to new and full moons^43, 49^, Corvina migrate from as far south as the Midriff Islands (Fig. 1) and form a FSA at one location in the estuaries of the Colorado River Delta (1–20 m depth) in the Northern Gulf of California, Mexico^43, 46^. During these brief yet predictable events, the FSA is targeted by a commercial gill net fishery of more than 500 small boats, which harvests several thousand tons of Corvina over the course of a few weeks^46^. Fishers locate the FSA through knowledge of their predictable migrations and by cueing on the courtship sounds produced by male Corvina during spawning (see Supplementary Fig. S1). The fishery is managed by a quota system in which estimates of catch per unit effort (CPUE) are used to estimate stock abundance and set catch levels for the spawning season. Despite high fishing pressure and uncertain sustainability^7, 43^ the stock size and status are largely unknown due to the lack of fisheries-independent data^50^. Therefore, the development of methods to estimate fish density from active acoustic measurements and sound levels would provide accurate estimates of fish abundance and biomass that could inform the quota system by reducing the probability of setting unsustainable harvest limits.

**Figure 2:**
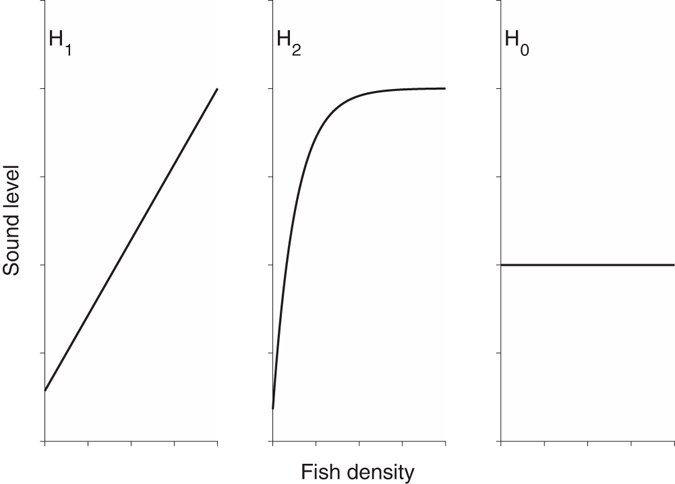
Hypotheses of the relationship between fish sound production levels and density. (H_1_) A strong, predictive linear relationship with increases in sound levels as a function of density, (H_2_) a weaker non-linear relationship where sound levels fail to respond to high densities, and (H_0_) a non-predictive relationship with changes in density not affecting sound levels.

We used active acoustic methods to measure density, lengths, abundance, biomass, and spatial extent of spawning fish and passive acoustic methods to measure the spatial distribution of sound levels attributable to fish sound production across eight surveys in March and April 2014. We then compared independent measures of fish density and sound levels at the FSA site to test the hypothesis that sound levels would increase linearly with density (H_1_, Fig. 2), resulting in a relationship with strong predictive power. While the potential for a weaker relationship that becomes asymptotic and insensitive at high densities exists (H_2_, Fig. 2), we expected that the careful selection of a sound level metric that is proportional to acoustic power would negate this possibility. We also acknowledged that a predictive relationship may not exist (H_0_, Fig. 2) due to the inherent variability in fish sound production rates at FSAs. However, we anticipated that a significant relationship would be observed if a period of stable sound production rate could be identified, resulting in a model to estimate fish density from sound production levels.

## Results

### Fish distribution, abundance, and biomass

**Figure 3:**
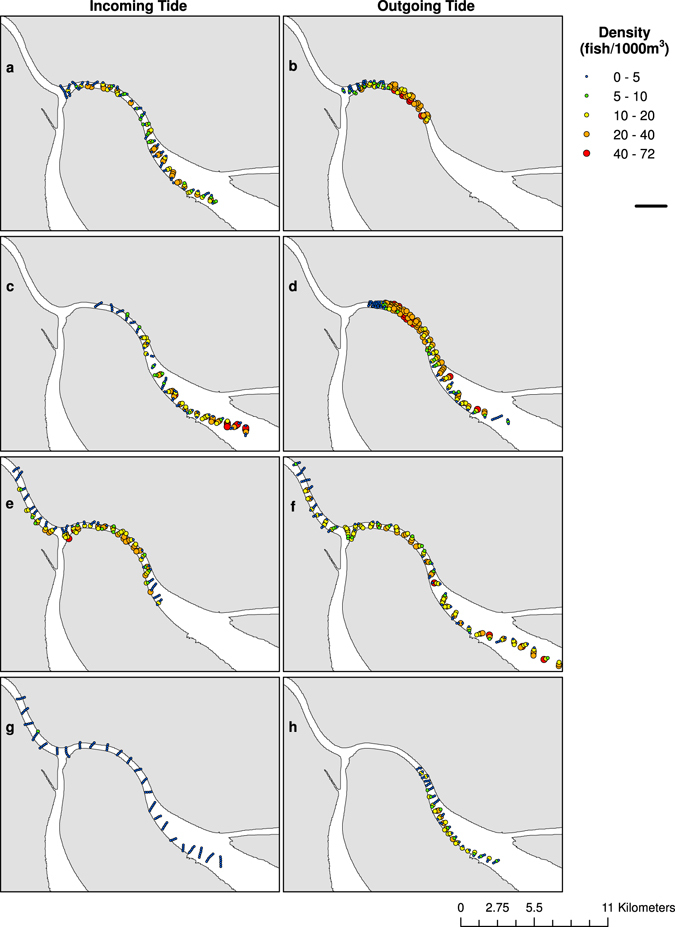
Spatial distribution of Gulf Corvina (*Cynoscion othonopterus*) densities (fish/1000 m^3^). Mean fish densities per every 150-m survey length in the spawning grounds of the northeastern channel of the Colorado River Delta on the incoming and outgoing tides on (**a**,**b**) 27 March 2014, (**c**,**d**) 28 March 2014, (**e**,**f**) 27 April 2014, and (**g**,**h**) 28 April 2014. All survey data are depicted, including 150-m survey lengths with densities of 0 fish/1000 m^3^. Collection of active acoustic data on the outgoing tide of 27 March 2014 (**b**) ended prior to the termination of passive acoustic data collection due to deteriorated survey conditions. Maps were generated using the ArcMap extension of ArcGIS version 10.2.2 (http://www.esri.com/, ESRI, USA).

Over the four days of active acoustic surveys, fish distributions varied between incoming and outgoing tides with higher densities extending progressively farther into the delta on the outgoing tide during spawning (Fig. 3). Differences in the density distributions mapped during the incoming and outgoing tides revealed that the aggregation moved slower than the survey progressed, mitigating the potential to survey individuals more than once. On the incoming tides of 27 March and 27 April 2014 and the outgoing tides of 28 March, 27 April, and 28 April 2014, we surveyed the entire aggregation present in the northeastern channel of the delta as evident from lower densities at the start and endpoints of surveys. Among these days, the aggregation was distributed over a linear distance of 8 to 25 km with densities greater than 5 fish/1000 m^3^. On the incoming tides on 28 March and 28 April and outgoing tide on 27 March 2014, the surveys did not sample the entire aggregation due to a miscalculation of the southern extent of the aggregation, absence of large Corvina in the channel, and degraded survey conditions, respectively. We observed large spawning events on all days except for 28 April 2014 when an imminent new moon prompted the aggregation to disperse from the reproductive grounds.

Mean densities (±S.E.) of Corvina ranged from 0.43 ± 0.07 to 15.97 ± 2.04 fish/1000 m^3^, which corresponded to an estimated 76,052 ± 12,380 to 1,551,729 ± 177,691 fish (±S.E.) across surveys (Table 2). Mean survey densities were higher on the outgoing tides in comparison to incoming tides, corresponding to the known timing of peak spawning activity, but were not significantly different (one-tailed t-test, p = 0.073). We estimated the abundance of Corvina in the northeastern spawning grounds at 1.53 million ± 196,443 and 1.55 million ± 177,691 fish (±S.E.) on the outgoing tides of 28 March and 27 April 2014 when we surveyed the entire aggregation in the northeastern channel. During the presence of FSAs, we observed fish of total lengths (*L*) 21–100 cm, as estimated from the following Kirchoff-ray Mode (KRM) model-derived relationship between mean lateral-aspect 120-kHz target strength (*TS*) and Corvina *L* (cm):

1 $$TS=22.44\,\mathrm{log}\,L-76.21;{r}^{2}=0.97$$

**Table 2:** Estimates of density and abundance of mature Gulf Corvina (*Cyonscion othonopterus*) per survey.

Tide	Volume (m^3^)	Density (fish/1000 m^3^)	S.E. (fish/1000 m^3^)	C.V. (%)	95% C.I. (fish/1000 m^3^)	Abundance (fish)	S.E. (fish)	95% C.I. (fish)
27 March 2014								
Incoming	91,966,129	7.47	0.99	13.3	5.86–9.95	686,987	91,046	538,922–915,063
Outgoing	49,576,934	13.04	1.87	14.3	9.32–16.66	646,483	97,709	462,057–825,952
28 March 2014								
Incoming	111,125,728	11.73	1.86	15.9	8.29–15.62	1,303,505	206,694	921,232–1,735,784
Outgoing	96,295,547	15.97	2.04	12.8	12.13–20.16	1,537,840	196,443	1,168,065–1,941,318
27 April 2014								
Incoming	132,894,113	11.00	1.21	11.1	8.60–13.37	1,461,835	160,802	1,142,889–1,776,794
Outgoing	153,181,587	10.13	1.16	11.5	8.18–12.98	1,551,729	177,691	1,253,025–1,988,297
28 April 2014								
Incoming	176,863,975	0.43	0.07	15.3	0.32–0.59	76,052	12,380	56,596–104,350
Outgoing	64,708,929	8.07	1.11	13.8	5.99–10.41	522,201	71,827	387,606–673,620

S.E. = standard error; C.V. = coefficient of variation (%); 95% C.I. = 95% confidence interval.

The results of the KRM model are detailed in Supplementary Fig. S2. The mean *L* (±95% C.I.) of the aggregated fish ranged from 38.677 ± 2.761 cm to 53.402 ± 1.407 cm among surveys, corresponding to mean weights (*W*) of 0.578–1.464 kg (Table 3). Biomass varied across days and tides depending on extent of area surveyed; however, on the outgoing tides of 28 March and 27 April 2014, estimated biomass was 2,145 ± 537 and 2,133 ± 480 metric tons (±95% C.I.), respectively (Table 3).

**Table 3:** Mean total length, weight, and estimated biomass of mature Gulf Corvina (*Cynoscion othonopterus*) per survey.

Tide	Length (cm)	S.E. (cm)	Weight (kg)	Biomass (kg)	S.E. (kg)	95% C.I. (kg)
27 March 2014						
Incoming	53.112	0.520	1.441	989,948	131,555	732,100–1,247,797
Outgoing	52.846	0.525	1.421	918,652	132,055	659,824–1,177,480
28 March 2014						
Incoming	51.442	0.568	1.315	1,714,109	272,461	1,180,086–2,248,132
Outgoing	52.512	0.338	1.395	2,145,287	274,386	1,607,491–2,683,082
27 April 2014						
Incoming	52.798	0.407	1.417	2,071,420	228,415	1,623,727–2,519,114
Outgoing	52.249	0.447	1.375	2,133,627	245,006	1,653,417–2,613,838
28 April 2014						
Incoming	38.677	1.409	0.578	43,958	7,333	29,586–58,331
Outgoing	53.402	0.718	1.464	764,502	105,656	557,417–971,588

S.E. = standard error; 95% C.I. = 95% confidence interval. Mean total length on the incoming tide of 28 April 2014 reflects the absence of high abundances of large Gulf Corvina within the survey area.

### Fish sound production and relationship to density

**Figure 4:**
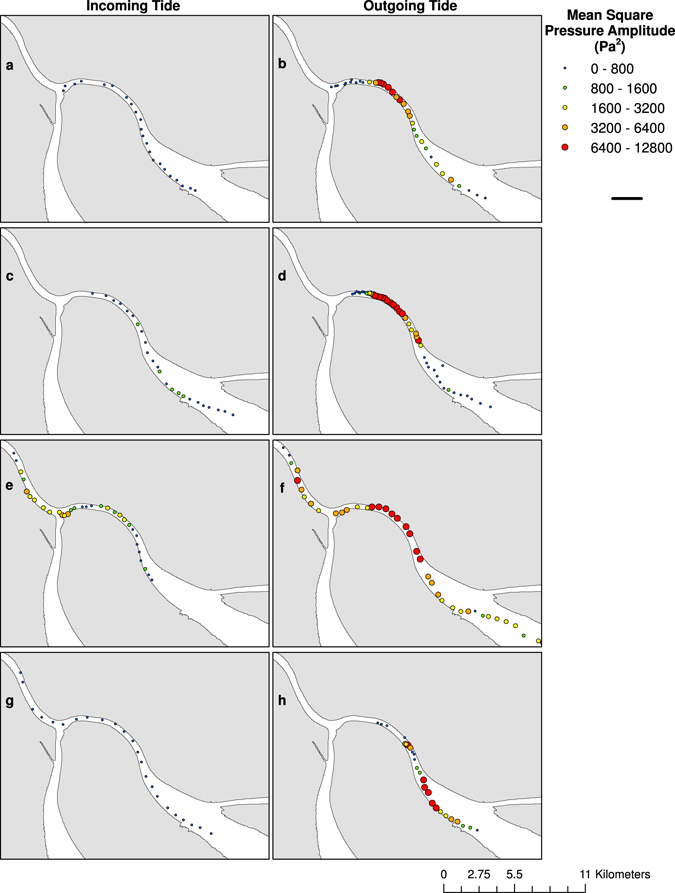
Spatial distribution of mean square pressure amplitude (*p* ^2^; Pa^2^; 251 Hz–498 Hz). Sound levels over the frequency band of Gulf Corvina (*Cynoscion* othonopterus) chorusing in the spawning grounds of the northeastern channel of the Colorado River Delta on the incoming and outgoing tides on (**a**,**b**) 27 March 2014, (**c**,**d**) 28 March 2014, (**e**,**f**) 27 April 2014, and (**g**,**h**) 28 April 2014. Collection of passive acoustic data on the outgoing tide of 27 March 2014 (**b**) continued after the conclusion of the active acoustic survey. Maps were generated using the ArcMap extension of ArcGIS version 10.2.2 (http://www.esri.com/, ESRI, USA).

We recorded Corvina sound production associated with spawning during all survey days. We did not observe sound production by any other fish species in our recordings. Measurements of mean square pressure amplitude (*p* ^2^) over the frequency band of Corvina chorusing (251–498 Hz, see Supplementary Fig. S1) ranged from 0.02 to 12,738 Pa^2^ among surveys but varied across the spawning grounds and tidal periods (Fig. 4). Values of mean *p* ^2^ per survey were significantly higher on the outgoing tides (one-tailed t-test, p < 0.001), coinciding with spawning; mean *p* ^2^ (±95% C.I.) ranged from 1.56 ± 1.9 Pa^2^ on the incoming tide of 28 April to 4430 ± 1004.0 Pa^2^ on the outgoing tide of 27 April 2014. Spatial differences in *p* ^2^ showed similarities to measured differences in fish density across the spawning grounds during the outgoing tides but not during the incoming tides (Figs 3 and 4). We observed elevated sound levels (*p* ^2^ > 800 pa^2^) attributable to dense aggregations and spawning activity over distances of 6.5 to 25 km on the outgoing tides of 27 and 28 March, and 27 and 28 April 2014.

**Figure 5:**
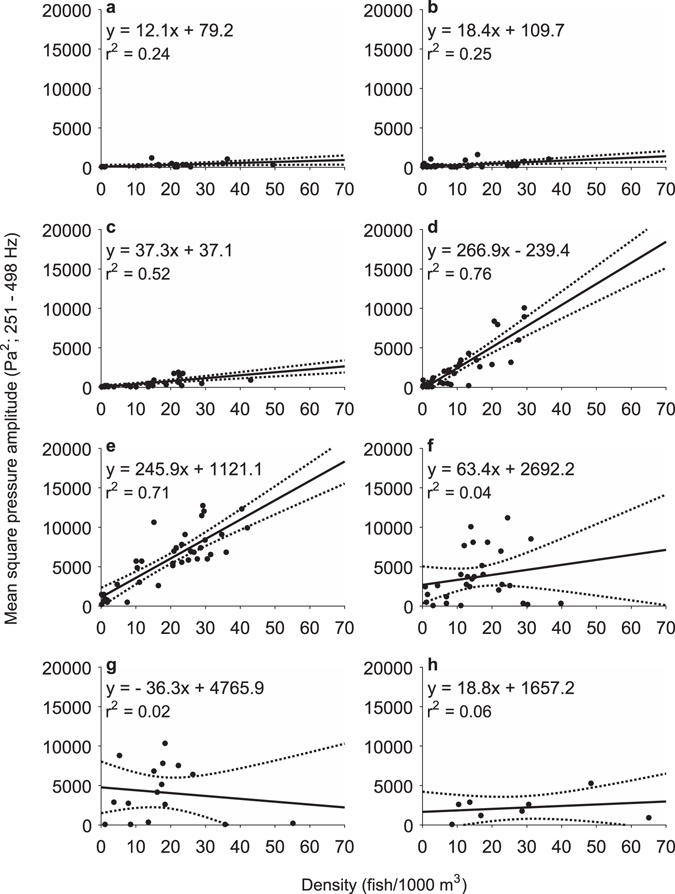
Relationship between sound levels and fish density over one-hour periods. Regressions of mean square pressure amplitude (*p* ^2^; Pa^2^; 251–498 Hz) versus fish density (*ρ*; fish/1000 m^3^) from (**a**) 3–2 hours before high tide, (**b**) 2–1 hours before high tide, (**c**) 1–0 hours before high tide, (**d**) 0–1 hours after high tide, (**e**) 1–2 hours after high tide, (**f**) 2–3 hours after high tide, (**g**) 3–4 hours after high tide, and (**h**) 4–5 hours after high tide. Dotted lines indicate 95% confidence intervals. Densities are mean densities per 150-m survey length that were nearest in space and time to sound measurements. See Supplementary Fig. S3 for hourly relationships using mean density per 300-m survey length.

**Figure 6:**
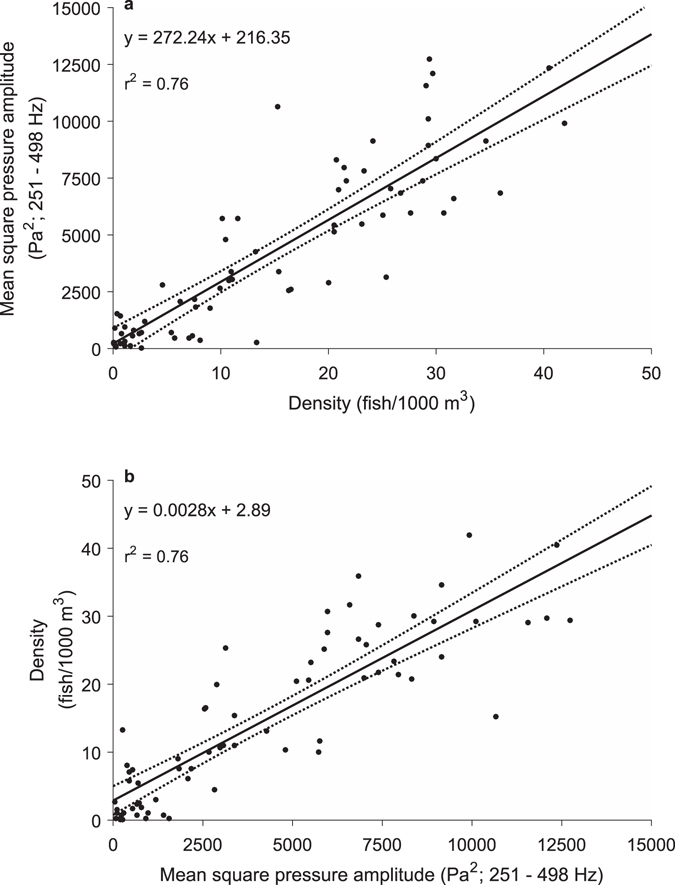
Relationship between sound levels and fish density during the peak spawning period. (**a**) Mean square pressure amplitude (*p* ^2^; Pa^2^; 251–498 Hz) as a function of density (*ρ*; fish/1000 m^3^) for measurements during the first two hours after high tide and (**b**) the modeled relationship generated for estimating *ρ* from future measurements of *p* ^2^, (F_1,68_ = 216, p < 0.001). Dotted lines indicate 95% confidence intervals. Densities are mean densities per 150-m survey length that were nearest in space and time to sound measurements. See Supplementary Fig. S4 for the modeled relationships using mean density per 300-m survey length.

Comparisons between *p* ^2^ and densities (*ρ*, fish/1000 m^−3^) resulted in different relationships dependent on the timing of measurements in relation to high tide (Fig. 5, ANCOVA, p < 0.001). The slopes of hourly regressions from 3 hours before high tide to high tide and from 2 to 5 hours after high tide were not significantly different (multiple comparisons, Tukey-Kramer, p > 0.52); however, these time periods were unsuitable to construct a model between *p* ^2^ and *ρ* due to the lack of good line fits and decoupling of changes in sound levels with density. From high tide until two hours after high tide, the slopes of regressions were homogeneous (multiple comparisons, Tukey-Kramer, p > 0.99) and significantly different from all other hours (multiple comparisons, Tukey-Kramer, p < 0.001), indicative of stationary call rates of Corvina that are additive in *p* ^2^ as a function of density during the two-hour window (Fig. 6). The modeled relationship between *ρ* and *p* ^2^ from high tide to two hours after high tide (Fig. 6) resulted in a method to estimate fish density from measured sound production (F_1,68_ = 216, p < 0.001) with the following equation:

2 $$\rho =0.0028{p}^{2}+2.89;\,{r}^{2}=0.76$$

This result did not refute our hypothesis that a relationship exists for a period of stable sound production (H_1_, Fig. 2). Equation (2) opens the possibility to estimate *ρ*, a fundamental measurement for population assessments, across different spatial scales (see Supplementary Figs S3 and S4) and thus abundance and biomass from future measurements of *p* ^2^ during the two hours after high tide.

## Discussion

The results of this study demonstrate that sound levels may be used to estimate the densities of soniferous fishes at FSAs, which provides an alternative means to assess their abundance, biomass, and spatial distribution. The selection of *p* ^2^ as an additive metric of Corvina sound production coupled with the identification of a two-hour period of stable sound production as a function of density resulted in the generation of a predictive relationship. While several methods are currently available to provide independent measurements fish density^12^ for comparison to sound levels, here we corroborate and further develop the use of active acoustic data to not only provide measures of density in a challenging shallow water environment but also to estimate abundance, mean length, biomass, and spatial distribution of fish. Our results support the continued use of passive acoustic methods to determine the spatio-temporal distributions of spawning activity and relative abundance^27, 29, 31, 34, 37^. More importantly, they show that when these methods are calibrated to the spatial and temporal dynamics of spawning activity of the study system, the difficulties of relating sound levels to actual fish densities can be overcome to provide a new fisheries-independent method to assess FSAs.

The selection and validity of different methods to estimate density for comparison to passive acoustic measurements are often challenged and limited by resource and equipment availability, environmental conditions, and spatial and reproductive dynamics of target species^12^; thus, different FSAs pose unique challenges that need to be considered. For example, aspects of the Corvina FSA present a series of obstacles that have thus far impeded fisheries-independent assessments: the aggregation forms in a remote shallow water environment (1–20 m) with near-zero visibility and large tidal ranges (7 m over a 6 hour period), which inhibits visual surveys; spawning takes place within a no-take biosphere reserve thereby discouraging invasive trawl sampling; and heavy fishing mortality during stock migrations and some spawning events within the protected reserve conflicts with mark-recapture, telemetry, and extractive methodologies. In this study, the versatility of active acoustic methods overcame these challenges to elucidate spawning and stock dynamics of Corvina and produce measurements of fish density, abundance, and biomass in a non-invasive manner.

Our active acoustic surveys generated the first fisheries-independent estimate of Corvina density, abundance, mean length, biomass, and spatial distribution of mature fish across four days of spawning in 2014 that may supplement existing catch per unit effort data to develop more robust and comprehensive stock assessments for the fishery. Although the active acoustic survey results elucidated the dynamics of the aggregation and the fishery, they were difficult to conduct due to sound propagation in shallow water environments, low insonified volumes, and potential vessel avoidance by fish^51, 52^. These challenges were mitigated by orienting the acoustic beam axis nominally 10° below horizontal to detect Corvina up to 50 m away from the vessel and removing the first 10 m range of acoustic data that exhibited low detection probability, respectively. As opposed to echo-integration^16^, fish were detected as tracks with multiple, spatially coherent *TS* measurements, permitting the calculation of density, abundance, and *TS* distributions of aggregated Corvina. The selection of Corvina-specific detection parameters and a lack of bycatch in the fishery^50^ provide credence that our estimates were not influenced by the presence of additional species. KRM modeling provided an equation to relate mean lateral-aspect *TS* to fish length for Corvina, thus enabling the estimation of mean fish lengths, weights, and total biomass.

While this study observed the formation of multiple FSAs comprised of over 1 million fish, these data do not implicitly provide an estimate of the total spawning population. To assess the total abundance and biomass of spawning populations using active or passive acoustics, the spatio-temporal dimensions and reproductive dynamics of FSAs need to be known. For example, previous efforts that have tracked fishing intensity at the FSA have shown that the Corvina likely aggregate in both channels of the Colorado River Delta^43^. As we only focused our efforts in the northeastern channel due to logistical limitations, only a portion of the spawning grounds were sampled. Additionally, FSAs of Corvina form prior to the new and full moons in the months of February to June, but it remains unknown whether the entire reproductive population participates in all spawning events with fixed residency times, if individuals spawn only once or multiple times, if mean length and weight across all spawning events are constant, and what are the effects of fishing mortality during migrations to the delta. However, with the future documentation of these dynamics, active and passive acoustic estimates can be calibrated using a hierarchical model to determine the total abundance and biomass of the spawning population^53^.

Fish sound production rates at FSAs are difficult to measure and highly variable, often influenced by the timing of spawning activity^29, 37, 38^, resulting in temporally inconsistent relationships between sound levels and abundance, as observed in this study. Consequently, the use of call numbers as predictors of fish abundance^35, 36^ has proven challenging for researchers^22, 23^. For many species, sound production associated with courtship and spawning is only present in males^26, 41^, thus without a knowledge of sex ratios, the use of call rates and occurrences to estimate abundance and density may only be applicable to males, assuming factors affecting sound production rates are understood. However, it has been argued that the call rates of courting males may be influenced by the presence of females, where call levels may be indicative of both male and female density^33^ but these relationships have not been fully investigated. At some FSAs high abundances of fish and sound production prevent the detection of individual calls altogether, as in this study, requiring measurements of ambient sound pressure levels^31, 54^ and the designation of indices to infer aggregation sizes^30, 32^.

Given these challenges and uncertainties, we compared fish sound production to independent measurements of density, which may be a more feasible manner to model their relationship at FSAs^22, 35^. Previous works have exemplified the potential of this method through comparisons of sound production indices with CPUE of simultaneous trawls^39^, densities of early stage eggs^31^, and asynchronous fish densities estimated with active acoustics^40^ and visual census^33^. In this study, we compared *p* ^2^ over the bandwidth of Corvina chorusing to fish density estimated simultaneously with active acoustics. We selected *p* ^2^ over other measurements, such as root-mean-square sound pressure level (dB), because the summed power from incoherent sounds from multiple calling fish is proportional to *p* ^2^. By comparing the relationship of *p* ^2^ with density over several one-hour periods, we were able to model the time-evolving relationship and identify patterns of similarity. The insensitivity of sound levels as a function of density prior to high tide and beyond two hours after high tide suggested inconsistencies in fish chorusing rates and a lack of coordinated courtship behaviors outside the peak spawning period. From high tide to two hours after high tide we observed a stable positive relationship, where increases in density corresponded linearly to increases in *p* ^2^, indicating a two-hour period of homogeneous sound production rates during which density can be estimated from passive acoustic measurements. Importantly, comparisons of regression slopes provided an approach to identify periods of stationary sound production, thereby negating the need to estimate calling and chorusing rates of males and sex ratios in a challenging environment. However, this strategy assumed that sex ratios remained stable across measurements, which has been estimated at 1:1 from fisheries landings^49^.

As with most assessment methods, the application of the model developed in this study for population monitoring and fishery management is limited by the inherent uncertainty of density estimates from sound levels, warranting confidence intervals and the potential for future improvement with additional data and possible sources of variance, such as tidal current strength. However, this model presents an opportunity to assess Corvina density and calculate abundance with confidence intervals, during the two hours after high tide, using passive acoustic survey techniques. With this relationship, sound production measurements can be used to monitor the spatio-temporal distribution of density and abundance and quickly detect potential impacts of fishing activities on stock size and spawning activity. Estimates of abundance can also be used to calculate biomass if fish lengths at the FSA site are known through independent sampling. Lengths may also be estimated using passive acoustics through analyses of the fundamental frequencies of sounds^55^.

Our results support the application of both active and passive acoustics to conduct surveys of FSAs that could provide needed information to resource managers, policymakers, conservationists, and fishing communities. We highlight the growing potential to develop comparative models between sound production and density of fishes using accurate independent measurements of fish density, but we acknowledge the future importance of testing their robustness against more complex acoustic modeling approaches with additional covariates that affect sound propagation, such as water depth, bathymetry, and boundary conditions^35^. Although our methods were applied to Corvina, they may be further developed and adapted to other commercially important species of soniferous fishes that form FSAs (Table 1), such as members of the cod, grouper, and croaker families^25, 29, 34^, based on knowledge of study species and systems and the appropriate spatial and temporal scales for predicting abundance from sound levels^35^. Active acoustic surveys can be designed to provide estimates of density, abundance, and biomass at FSAs; however, other independent sources of density may be viable, such as visual census, if conditions permit. The inclusion of fixed, long-term acoustic recorders in future efforts at FSAs will enable entire spawning grounds to be assessed simultaneously over multiple spawning events, providing a cost effective means to capture ambient sound and estimate abundance across a suite of habitats and economic environments. The continuation, improvement, and expansion of these methods across multiple species will validate passive acoustics as a frontier tool for the management and conservation of fish populations.

## Methods

All methods were conducted in accordance with approved guidelines and regulations. The use of deceased fish from the fishery for *TS* modeling and collection of acoustic data were approved by the Institutional Animal Care and Use Committee (IACUC) at the University of California San Diego under IACUC protocol S13240 and at the University of Texas at Austin under IACUC protocol AUP-2015-00162.

### Survey design

Prior to the new moons in March and April 2014, we simultaneously conducted eight active and passive acoustic surveys in the northeastern channel of the Colorado River Delta from two small (8 m long) fishing vessels. As there are only two days of peak spawning per FSA event (2–3 days before new and full moons)^43^, we focused our efforts on these days to assess the aggregation and minimize daily variation. We surveyed the spawning grounds of Corvina twice daily, on the incoming and outgoing tide, progressing in the direction of tidal current (see Supplementary Fig. S5 and Table S1). Active acoustic surveys of Corvina were comprised of semi-randomized parallel transects across the delta channel spaced, on average, 0.5 km apart with deviations attributable to tidal flow and avoidance of surface gill nets. From a second vessel, we conducted passive acoustic surveys of Corvina sound production in synchrony with the timing and location of each active acoustic transect (see Supplementary Fig. S5), and the independent measurements were compared to estimate the spatial distribution of sound levels attributable to spawning Corvina and to model the relationship between acoustic measurements of density and sound production.

### Active acoustic sampling

We conducted active acoustic sampling with a 120-kHz echosounder (ES60, Simrad-Kongsberg, Norway) configured with a split-beam transducer (ES120-F; Simrad-Kongsberg, Norway) having a nominal 9° beam width. Prior to sampling, we calibrated the complete system using the standard sphere method^56, 57^ and a 38.1 mm diameter sphere constructed of tungsten carbide with 6% cobalt binder material (Bal-tec^TM^, Micro Surface Engineering, Inc., USA). For each survey, we collected water temperature and salinity profiles using a handheld CTD (Castaway®-CTD, SonTek/Xylem, Inc., USA) to measure sound speeds, calculate absorption coefficients, and calibrate data during processing. Throughout the surveys, the echosounder operated with a pulse duration of 256 µs, a ping rate of 0.25 s per transmission, and a transmit power of 200 W. The transducer was positioned off the port side of the vessel with the face nominally 0.53 m below the sea surface. As the survey area ranged in depth from 1 to 20 m, the beam axis was oriented nominally 10° below horizontal to increase the insonified volume, limit reverberation from the water surface, and increase the observational range. Surveys were performed at an average speed of 6 knots, and received power and split-beam phase data, indexed by time and geographic location, were sampled and stored every 64 µs.

### Passive acoustic sampling

We recorded ambient sound at a single random position along each active acoustic transect (see Supplementary Fig. S5), using a calibrated Tascam DR-680 Portable Multitrack Recorder (TEAC Corporation, Japan) and a single HTI-96-MIN hydrophone (High Tech, Inc., USA; sensitivity = −192.0 dBV/µPa). The location of a recording along each transect was dictated by ability of the vessel to position itself along the next transect at the same time as the active acoustic vessel passed by. Files were digitized with 24-bit resolution at a sampling rate of 192 kHz and stored (.wav format) on a secure digital high capacity (SDHC) memory card. At each sampling location, the vessel engine was turned off, and 60 s of ambient sound was recorded with the hydrophone lowered 2 m below the hull of the vessel as the vessel drifted across the path of the portion of the active acoustic transect recently completed.

### Specimen collection and target strength modeling

To identify Corvina in the active acoustic dataset and estimate mean *L* and *W*, we collected five individuals from the local fishery and used measurements of their shapes and *L* values (27.4–75.3 cm) to parameterize the Kirchoff-ray Mode model^58^ (KRM) and estimate their target strength (*TS*) values at 120 kHz. The range of *L* approximated the size range (*L* = [21, 100 cm]) of mature Corvina at the FSA^46, 49, 50^. Each specimen was photographed, weighed, measured, and stored on ice before radiography. We radiographed fish ventrally and laterally at a distance of 116 mm with 48 kV at 15 mAs using a MinXray HF100+ mobile X-ray unit (MinXray, Inc., USA). Fish bodies and swim bladders were traced from X-ray images and used to parameterize the KRM. We estimated the average lateral-aspect *TS* for each length by summing the backscattering cross sections across incident angles of 65° to 115° (90° equals broadside), dividing by 180°, then converting to decibels^58^. The relationship between *TS* at 120 kHz and Corvina *L* (cm) was determined by a nonlinear least squares fit of the following equation:

$$TS=m\,\mathrm{log}\,L+b$$

### Active acoustic data analysis

Active acoustic data were calibrated, visualized, and processed using commercial software (Echoview V5.4, Echoview Software Pty Ltd, Australia). We identified seabed echoes using an automatic detection algorithm and manual editing, and excluded them from further analyses. Echoes within the near-field range (0.51 m), corresponding to a beam-axis depth of 0.62 m, were also removed before further processing. We visually examined echograms to identify and remove regions of noise, propeller wash, bubbles, and along-shore segments of the surveys, resulting in a series of noise reduced across-channel transects for each survey. We identified individual targets attributable to Corvina using a single target detection operator (Split Beam Method 2, Echoview Software Pty Ltd, Australia) and *TS* and angular-position operands. A conservative, minimum-*TS* threshold of −46.5 dB was chosen, based on the mean KRM-*TS* versus *L* (see Supplementary Fig. S2), to include measures of all sizes of mature individuals potentially within the spawning aggregation^49, 50^. We refined the parameters of single target detection after the completion of sensitivity analyses (see Supplementary Table S2). To identify individual fish from multiple single targets, fish tracks were detected using a tracking algorithm (Alpha-Beta, Echoview Software Pty Ltd, Australia) parameterized with limits on range, alongships- and athwartships-angles, and time. We required a minimum of two single targets to generate a track and adjusted additional parameters for proficiency based on vessel speed and transducer orientation (see Supplementary Table S2).

We gridded fish track detections into 1-m range bins and exported with summed wedge volume^59^ (m^3^) per bin to estimate the probability density function (PDF) of fish density versus distance from the transducer face and depth. Regions of data corresponding to ranges less than 10 m (2.3 m depth) from the transducer were excluded from further analyses, as a result of exhibiting a non-stationary density PDF attributed to low beam volume, fish avoidance of the vessel, or both^16, 52^; beyond 10 m range (2.3 m depth) fish densities were stationary and homogenous. We separately partitioned the remaining data (range 10 m to the seabed) into 150-m and 300-m survey lengths and complete across-channel transects and exported them with summed wedge volume, total number of fish tracks, and mean geographic position and time per partition. We estimated fish density (fish/1000 m^3^) for each 150-m and 300-m survey length and complete across-channel transect by dividing the total number of fish tracks by summed wedge volume. The spatial distributions of fish densities were visualized in geographic information system software (ArcMap, Esri, USA).

Mean fish density per survey was calculated by a transect-volume weighted average of across-channel transect densities. Standard errors, coefficients of variation, and 95% confidence intervals (95% C.I.) of survey densities were estimated from bootstrap resampling (n = 10,000) of mean density of across-channel transect densities^16, 17^. We estimated fish abundance per survey by multiplying mean fish density by total survey volume as determined from bathymetric data and time-evolving tidal height. Standard errors and 95% C.I. of abundance were estimated by multiplying the bootstrap-estimated values for density by the volume of surveys. Autocorrelation analysis was conducted to ensure statistical independence between across-channel transect densities and the unbiased estimation of variance^17^. However, for systematic surveys with strong spatial trends in fish densities (e.g., aggregated or correlated), variance can be biased high, potentially warranting post-stratification of data to mitigate variance inflation^17, 60^; this potential bias was not evaluated in this study. We tested that mean density on incoming tides was lower than outgoing tides with a one tailed t-test (α = 0.05) after the data were tested for homoscedasticity with Levene’s test (α = 0.05) and normality with an Anderson-Darling test (α = 0.05).

To estimate mean fish length and biomass for each survey, we isolated mean *TS* of fish tracks with at least three consecutive measures to increase the probability of detections with multiple incidence angles. We converted mean *TS* of fish tracks to *L* using the derived KRM-*TS* versus *L* equation, and mean *L* per survey was calculated. We converted mean *L* to mean weight using a length-to-weight relationship previously estimated for Corvina^49, 50^ and multiplied by total abundance to estimate spawning stock biomass per survey. Variances of mean *L* and abundance were propagated and summed in quadrature as fractional uncertainties to estimate the standard errors and 95% C.I. of biomass.

### Passive acoustic data analysis

We visually and audibly inspected recordings of ambient sound and extracted 20-s segments free of nearby boat noise and operational disturbances. For each 20-s recording, the mean square pressure amplitude (*p* ^2^; Pa^2^) over the 251–498 Hz band was measured by integrating the pressure spectral density (µPa^2^/Hz; Hanning window; 16384-point FFT) across the peak frequency bandwidth of Corvina chorusing (see Supplementary Fig. S1), thereby limiting contributions from nearby vessels. We plotted *p* ^2^ at each sampling location in geographic information system software (ArcMap, Esri, USA) to visualize the spatial and temporal distribution of sound production and their relation to fish densities. We tested that mean *p* ^2^ on the incoming tide was lower than on outgoing tides with one-tailed t-test (α = 0.05) after the data were tested for homoscedasticity with Levene’s test (α = 0.05) and normality with an Anderson-Darling test (α = 0.05).

### Comparison of active and passive acoustic data

Fish densities per 150-m and 300-m survey length (*ρ*; fish/1000 m^3^) that were nearest in space and time to each passive acoustic recording station were compared to *p* ^2^ measurements separately. Measurements of *ρ* and *p* ^2^ that were not coupled in space and time were excluded from comparisons. We generated and examined plots of *p* ^2^ vs. *ρ* as a function of time in relation to high tide to test our hypotheses that a relationship with either strong (H_1_, Fig. 2) or weak (H_2_, Fig. 2) predictive power exists among measurements. We compared *p* ^2^ and *ρ* over one hour periods (3 hrs before high tide – 5 hrs after high tide) to observe the evolving relationship between sound production and fish density. We generated regressions between *p* ^2^ vs. *ρ* for each hour using generalized linear model (GLM) regressions. We compared regression slopes using analysis of covariance (ANCOVA, α = 0.05) to test for homogeneity and through multiple comparisons (Tukey-Kramer Method, α = 0.05) to identify the time period with a stable relationship between *p* ^2^ and *ρ*. We combined hour periods with homogeneous regression slopes to test our hypotheses and construct a model (GLM) to estimate *ρ* from *p* ^2^. Results from complementary analyses using densities over 150-m and 300-m survey lengths were compared and tested for homogeneity using analysis of covariance (ANCOVA, α = 0.05) and found to not be significantly different; thus, comparative results are only presented for modeled relationships between *p* ^2^ and *ρ* per 150-m survey length (see Supplementary Figs S3 and S4 for a detailed description of 300-m results).

## Electronic supplementary material

Supplementary Information for Rowell et al. Estimating fish abundance at spawning aggregations from courtship sound levels
