# Supplementary material for: Estimating fish abundance at spawning aggregations from courtship sound levels
Source: Sci Rep. 2017 Jun 13;7:3340. doi: 10.1038/s41598-017-03383-8 (PMC5469787; doi:10.1038/s41598-017-03383-8)
Supplement: Supplementary file 1 — Supplementary Information for Rowell et al. Estimating fish abundance at spawning aggregations from courtship sound levels [file 41598_2017_3383_MOESM1_ESM.pdf]

Supplementary Information

Estimating fish abundance at spawning aggregations from courtship sound levels

Timothy J. Rowell[1,*], David A. Demer[2], Octavio Aburto-Oropeza[1], Juan José Cota-Nieto[3], John R. Hyde[2] & Brad E. Erisman[4]

[1]Marine Biology Research Division, Scripps Institution of Oceanography, University of California San Diego, 9500 Gilman Drive, La Jolla, California 92093 USA. [2]NOAA Fisheries, Southwest Fisheries Science Center, 8901 La Jolla Shores Drive, La Jolla, California 92037-1508 USA. [3]Centro Para la Biodiversidad Marina y Conservación A.C., Calle del Pirata, # 420, Fracción Benito Juárez, La Paz, Baja California Sur 23090 México. [4]Marine Science Institute, The University of Texas at Austin, 750 Channel View Drive, Port Aransas, Texas 78373 USA. Correspondence and requests for materials should be addressed to T.J.R. (email: trowell@ucsd.edu)

Supplementary Figures

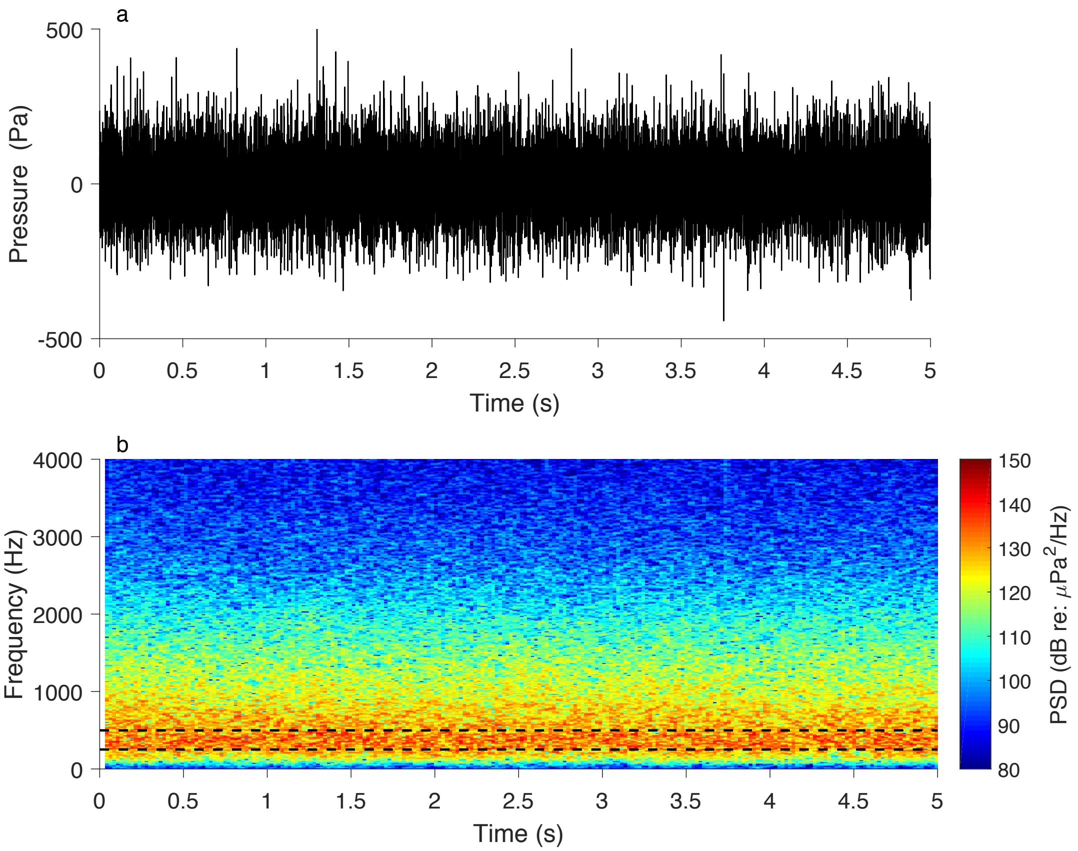

Supplementary Figure S1. Oscillogram and spectrogram of a period of sustained Gulf Corvina (*Cynoscion othonopterus*) chorusing. (a) An oscillogram of a 5-s period of ambient sound recorded at the spawning grounds in the Colorado River Delta, depicting the high amplitudes (Pa) of sound produced by chorusing. (b) A spectrogram of ambient sound over the same 5-s period. The frequency bandwidth of Gulf Corvina chorusing is evident by higher amplitudes of PSD (pressure spectral density). The bandwidth (251 – 498 Hz) over which mean square pressure amplitude (p^2) was integrated is indicated by the dashed black line.

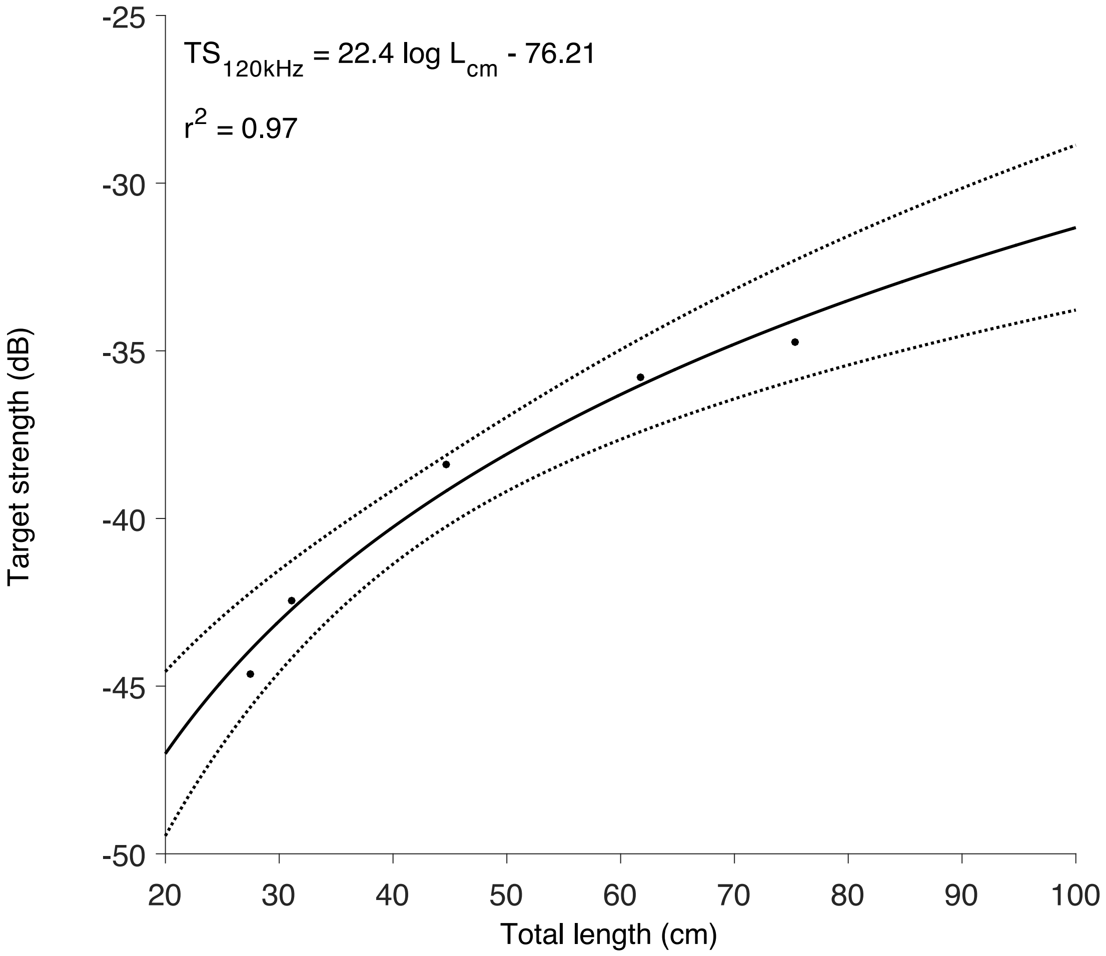

Supplementary Figure S2. Kirchoff-ray Mode (KRM) model-derived relationship between mean lateral-aspect 120-kHz target strength (*TS*) and Gulf Corvina (*Cynoscion othonopterus*) total length (*L*; cm). The model was fitted using a range of *L* of Corvina known to be present at the fish spawning aggregation site. The resulting model parameters are comparable to the reported values for other physoclistous fishes, see *Simmonds J. & MacLennan D. Fisheries Acoustics: Theory and Practice (eds. Simmonds, J. & MacLennan, D.) 1-437 (Blackwell Science Ltd, 2005)*. While the relationship and estimation of variance may be improved with additional data, the model was concluded to be adequate as a tertiary result used within the methodology due to the alignment of parameter estimates with reported values coupled with a high coefficient of determination ($r^2 = 0.97$). The model was used to select a conservative threshold of *TS* for single target detection and estimate the mean *L* of Corvina in survey areas. Dotted lines indicate 95% confidence intervals.

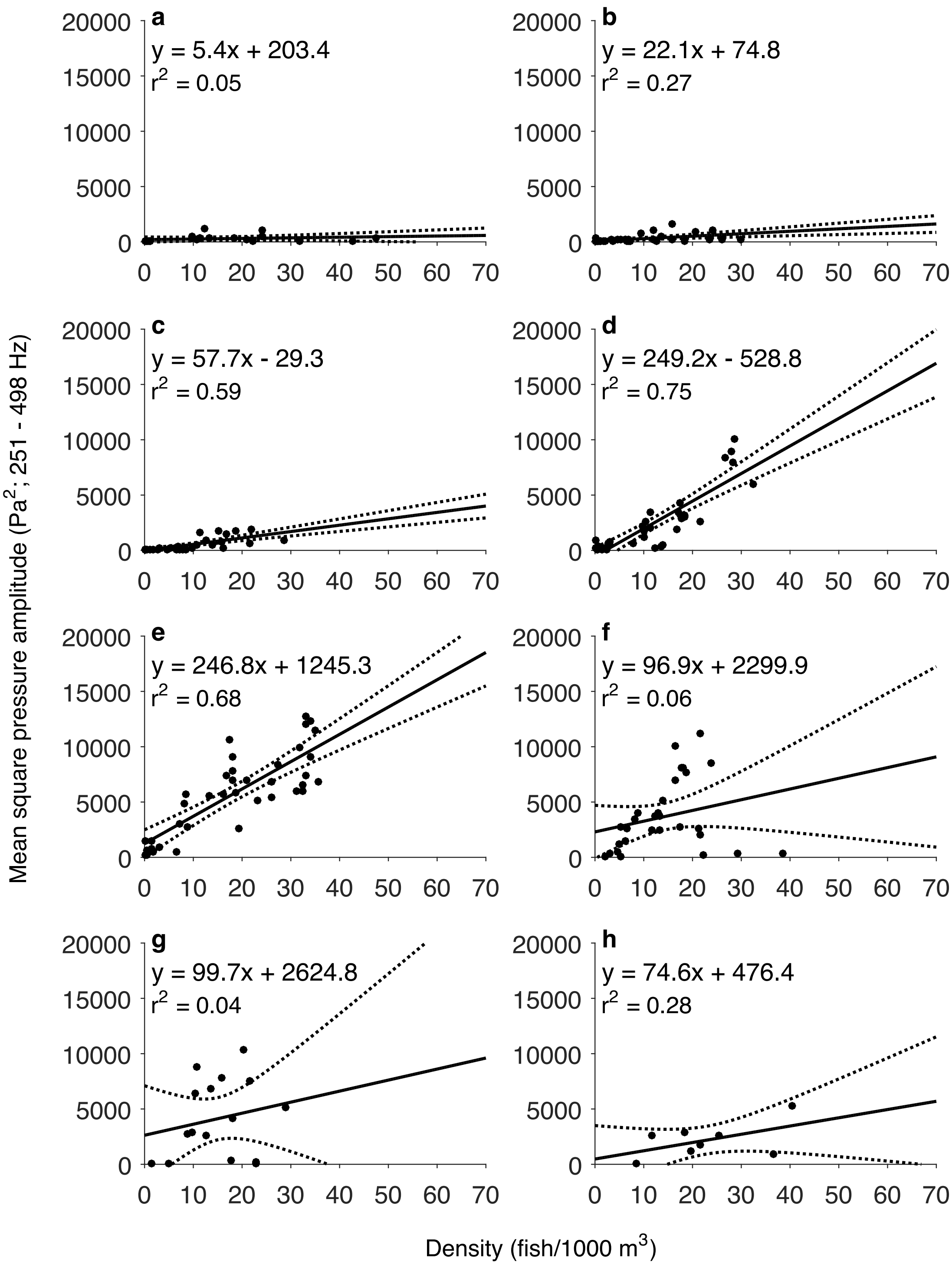

Supplementary Figure S3. Relationship between sound levels and fish density per 300-m survey length over one-hour periods. Regressions of mean square pressure amplitude (p^2; Pa^2; 251 – 498 Hz) versus fish density (ρ; fish/1000 m^3) from (a) 3 – 2 hours before high tide, (b) 2 – 1 hours before high tide, (c) 1 – 0 hours before high tide, (d) 0 – 1 hours after high tide, (e) 1 – 2 hours after high tide, (f) 2 – 3 hours after high tide, (g) 3 – 4 hours after high tide, and (h) 4 – 5

hours after high tide. Dotted lines indicate 95% confidence intervals. Densities are mean densities per 300-m survey length that were nearest in space and time to sound measurements. The slopes of hourly regressions between p^2 and densities per 300-m transect lengths (ρ; fish/1000 m^{-3}) were significantly different (ANCOVA, $p < 0.001$). The slopes of hourly regressions from 3 hours before high tide to high tide and from 2 to 5 hours after high tide were not significantly different (multiple comparisons, Tukey-Kramer, $p > 0.62$); however, these time periods were unsuitable to construct a model between p^2 and ρ due to the lack of good line fits and decoupling of changes in sound levels with density. From high tide until two hours after high tide, the slopes of regressions were homogeneous (multiple comparisons, Tukey-Kramer, $p > 0.99$) and significantly different from all other hours (multiple comparisons, Tukey-Kramer, $p < 0.04$) except for comparisons between 0 – 1 and 3 – 4 hours after high tide (multiple comparison, Tukey-Kramer, $p = 0.35$), 0 – 1 and 4 – 5 hours after high tide (multiple comparison, Tukey-Kramer, $p = 0.15$), 1 – 2 and 3 – 4 hours after high tide (multiple comparison, Tukey-Kramer, $p = 0.42$), and 1 – 2 and 4 – 5 hours after high tide (multiple comparison, Tukey-Kramer, $p = 0.18$).

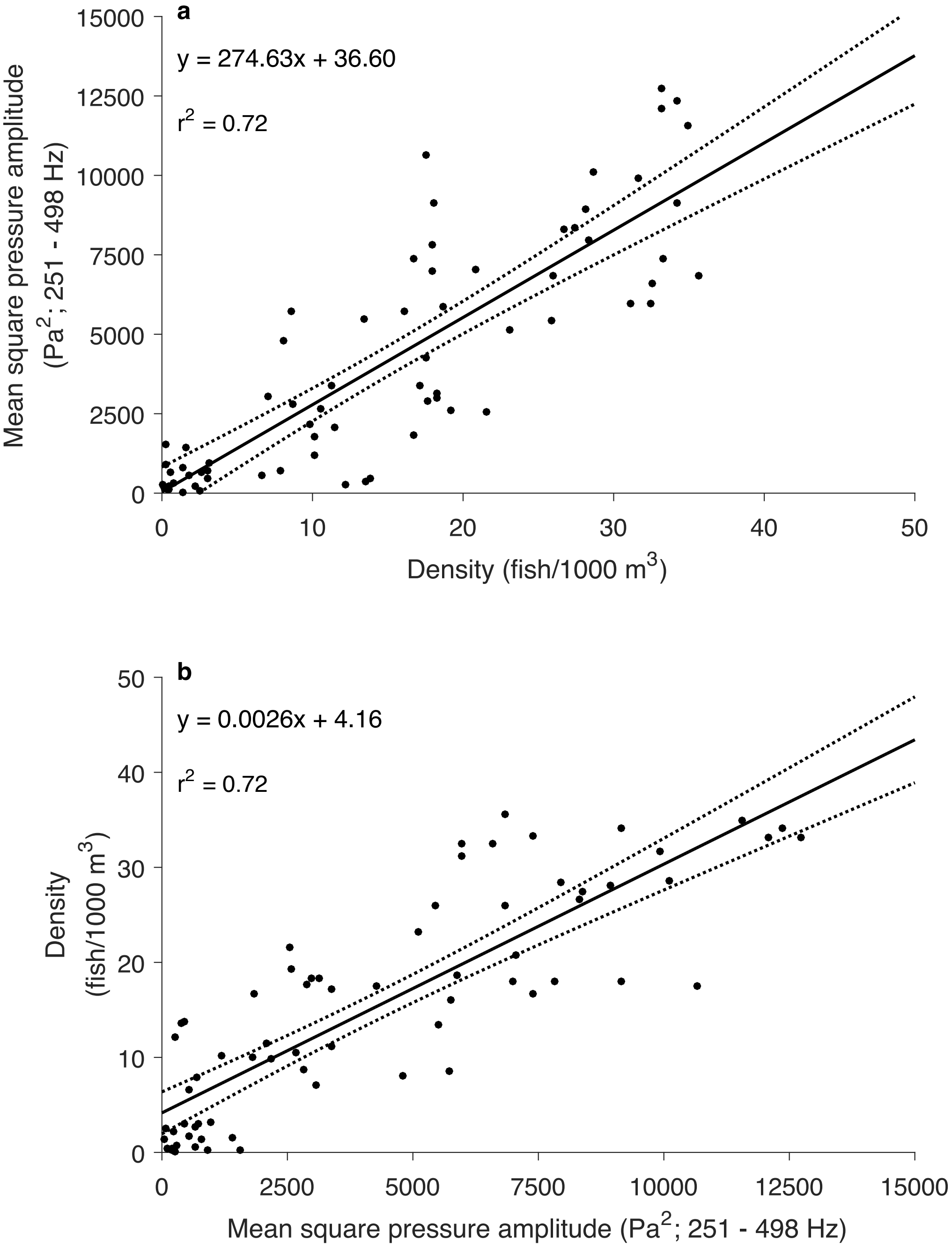

Supplementary Figure S4. Relationship between sound levels and fish density per 300-m survey length during the peak spawning period. Mean square pressure amplitude (p^2; Pa^2; 251 – 498 Hz) as a function of density (ρ; fish/1000 m^3) for measurements during the first two hours after high tide and (b) the modeled relationship generated for estimating ρ from future measurements of p^2, (GLM; $F_{1,68} = 174$, $p < 0.001$). Dotted lines indicate 95% confidence intervals. Densities are mean densities per 300-m survey length that were nearest in space and time to sound measurements. The slopes of regressions between p^2 and densities per 150-m and per 300-m survey length were not significantly different (ANCOVA, p = 0.93), indicating a stable relationship across two different spatial scales.

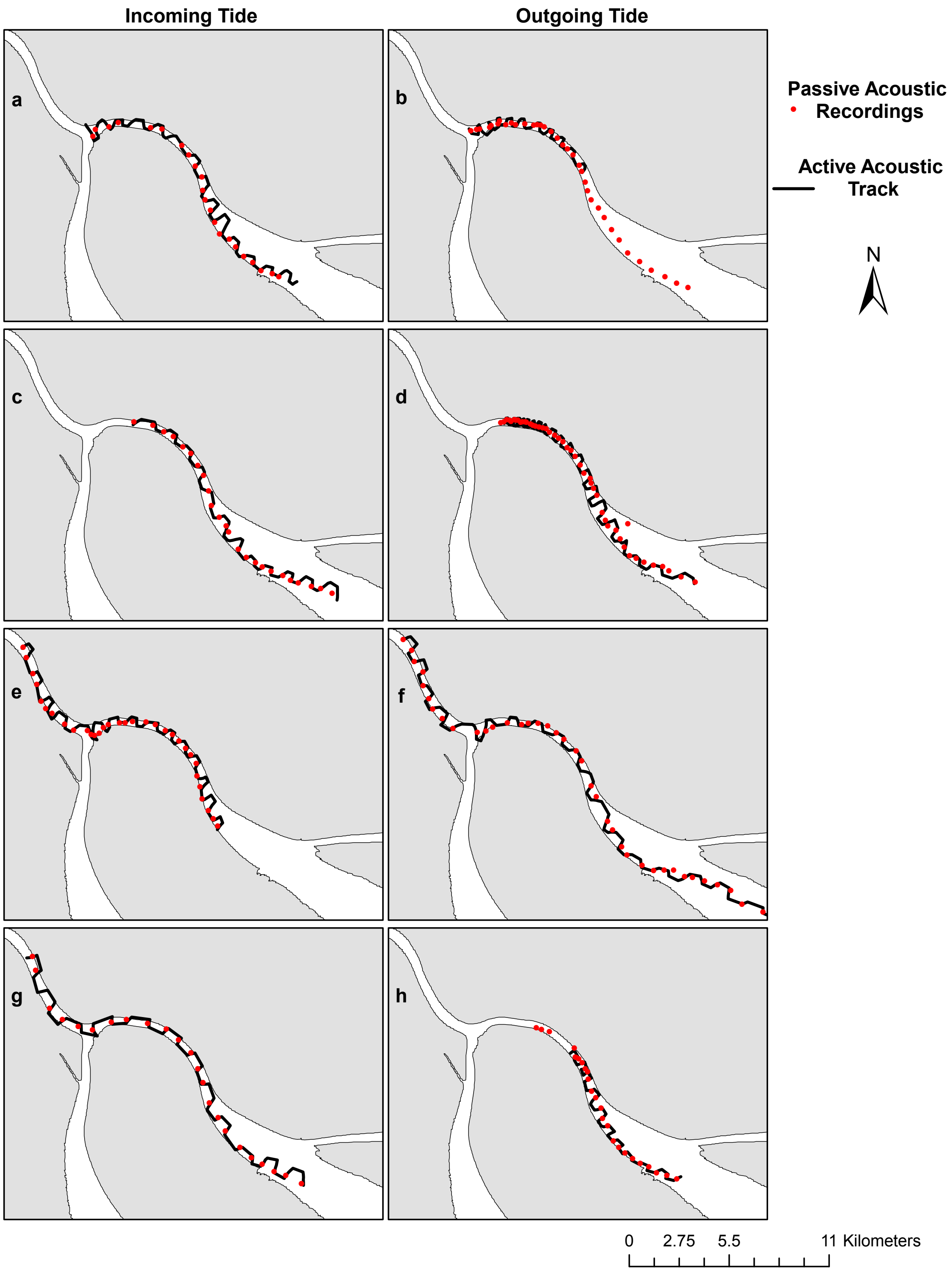

Supplementary Figure S5. The locations of passive acoustic recordings and vessel tracks of active acoustic surveys. The two vessels worked in coordination to survey the spawning grounds of the northeastern channel of the Colorado River Delta on the incoming and outgoing tides on (a, b) 27 March 2014, (c, d) 28 March 2014, (e, f) 27 April 2014, and (g, h) 28 April 2014. On average one passive acoustic recording was made per across-channel transect

completed by the vessel conducting active acoustic sampling. Passive acoustic recordings were made as the vessel drifted over the tracks of active acoustic transects. Passive and active acoustic measurements that were not coupled in space and time were not included in comparative analyses and model generation. Active acoustic sampling ended prior to the completion of passive acoustic sampling on the outgoing tide of 27 March 2014 (b) due to aberrant survey conditions. Maps were generated using the ArcMap extension of ArcGIS version 10.2.2 (http://www.esri.com/, ESRI, USA).

Supplementary Tables

Table S1. The start and end times of active acoustic surveys. Times are provided as Greenwich Mean Time (GMT) and hours in relation to high tide (re: high tide). Negative values signify hours prior to high tide, while positive values signify hours after high tide. Time at high tide was determined from tidal predictions for El Golfo de Santa Clara, Sonora, Mexico. *Next calendar day (GMT).

Tide	Start Time (GMT)	Start Time (re: high tide)	End Time (GMT)	End Time (re: high tide)
27 March 2014				
Incoming	16:52	-2.4	19:02	-0.23
Outgoing	19:02	-0.23	21:04	1.8
28 March 2014				
Incoming	17:10	-2.73	19:12	-0.7
Outgoing	19:40	-0.23	23:22	3.47
27 April 2014				
Incoming	18:46	-1.50	21:12	0.93
Outgoing	21:52	1.60	00:56*	4.67
28 April 2014				
Incoming	18:12	-2.67	20:46	-0.10
Outgoing	22:06	1.23	23:38	2.77

Table S2. Thresholds and parameters used to detect single targets (Split Beam Method 2, Echoview V5.4, Echoview Software Pty Ltd, Australia) and fish tracks (Alpha-Beta, Echoview V5.4, Echoview Software Pty Ltd, Australia).

Parameter	Value
Single Target Detection	
TS threshold (dB)	-46.5
Pulse length determination level (dB)	13.0
Minimum normalized pulse length	0.6
Maximum normalized pulse length	5.0
Beam compensation model	Simrad LOBE
Maximum beam compensation (dB)	12.0
Maximum standard deviation of minor and major axis angles (°)	1.5
Fish Track Detection	
Minimum number of single targets in track	2.0
Minimum number of pings in track (pings)	1.0
Maximum gap between single targets (pings)	3.0
Major axis weight	30.0
Minor axis weight	30.0
Range weight	40.0
TS weight	0.0
Ping gap weight	0.0
Alpha	0.7
Beta	0.5
Major and minor axis exclusion distance (m)	4.0
Range exclusion distance (m)	0.4
